# P06 The use of procalcitonin testing to improve antibiotic stewardship in all cause respiratory admissions: a retrospective analysis

**DOI:** 10.1093/jacamr/dlac053.006

**Published:** 2022-05-31

**Authors:** S Paterson, K Carey, E M Murray, E McCarron, P Rafferty, B Smyth, A Brady, G McKeeman, K Ryan, J Kidney, G Ong

**Affiliations:** 1 Microbiology Department, Kelvin Building, Royal Victoria Hospital, Belfast Health and Social Care Trust, Belfast, UK; 2 Biochemistry Department, Kelvin Building, Royal Victoria Hospital, Belfast Health and Social Care Trust, Belfast, UK; 3 Department of Reparatory Medicine, Mater Infirmorum Hospital, Belfast Health and Social Care Trust, Belfast, UK; 4 Pharmacy Departments, Mater Infirmorum Hospital and Royal Victoria Hospital, Belfast Health and Social Care Trust, Belfast, UK

## Abstract

**Background:**

Improving antibiotic stewardship whilst simultaneously optimizing patient safety is a perpetually vexing clinical conundrum, which has been compounded by the current COVID-19 pandemic. Procalcitonin (PCT) measurement has previously demonstrated utility in this regard, when combined with routine clinical investigation, in certain patient populations.

**Objectives:**

To assess whether the inclusion of PCT measurement as part of routine clinical care, instituted during a quality improvement project (QIP), increases the appropriateness of antibiotic administration.

**Methods:**

A retrospective analysis was performed on 6 month interim data obtained from May to October 2021 during a QIP, which assessed the effect of PCT measurement on antimicrobial stewardship. All patients included had a primary diagnosis of respiratory illness and were analysed both together and as COVID-19 and non-COVID-19 subgroups to assess how often antibiotics were commenced on admission, duration of treatment and appropriateness of use. Finally, as sending microbiological samples made up part of the protocol, sample sending frequency was also studied.

**Results:**

Thirty patients were included in both the COVID-19 and non-COVID-19 baseline subgroups who did not have PCT testing performed. Fifty-two patients were included in the PCT subgroup (27 COVID-19 positive and 25 COVID-19 negative). Following introduction of PCT testing, commencement of antibiotics on admission was reduced overall and in the COVID-19 positive subgroup (*P* = 0.0426 and *P* = 0.0446, respectively) with a significant decrease in inappropriate antibiotic prescribing in these two groups (*P* = 0.011 and *P* = 0.0157, respectively) and a trend towards reduced prescribing of AWaRe watch group antibiotics such as ceftriaxone. However, once prescribed, there was no difference in duration of antibiotic treatment or the frequency of microbiological sampling.

**Conclusions:**

The data from this interim data analysis demonstrate that PCT measurement, when combined with routine clinical investigations in the acute respiratory setting, can be used to reduce inappropriate antibiotic prescribing. This was significantly reduced overall and in the COVID-19 positive subgroup but lost statistical significance in the COVID-19 negative subgroup, where it could be hypothesized that heterogeneity and inclusion of respiratory diseases where PCT has previously encountered difficulty in determining the presence of acute bacterial infection may be the cause. The significant effect demonstrated in the COVID-19 positive subgroup suggests particular utility in this patient population.

